# Vitamin D deficiency is a potential risk factor for lipid Amphotericin B nephrotoxicity

**DOI:** 10.1371/journal.pntd.0007567

**Published:** 2019-07-11

**Authors:** Daniela Ferreira, Ana Carolina de Bragança, Rildo Aparecido Volpini, Maria Heloisa Massola Shimizu, Pedro Henrique França Gois, Adriana Castello Costa Girardi, Antonio Carlos Seguro, Daniele Canale

**Affiliations:** 1 Laboratorio de Investigacao Medica 12 (LIM12), Faculdade de Medicina, Universidade de São Paulo, São Paulo, Brazil; 2 Laboratorio de Investigacao Medica 12 (LIM12), Hospital das Clinicas HCFMUSP, Faculdade de Medicina, Universidade de São Paulo, São Paulo, Brazil; 3 Laboratorio de Cardiologia Genetica e Molecular, Instituto do Coracao, Universidade de São Paulo, São Paulo, Brazil; Faculty of Science, Ain Shams University (ASU), EGYPT

## Abstract

Invasive fungal infections (IFI) is a worldwide serious health problem and Amphotericin B (AmB) has been considered the drug of choice for IFI treatment. Despite its efficacy, clinical use of AmB has been associated with renal toxicity. Some lines of evidence have shown that an extemporaneous lipid emulsion preparation of AmB (AmB/LE) was able to attenuate nephrotoxicity, presenting similar benefits at a lower cost. Studies have been demonstrating that hypovitaminosis D may hasten the progression of kidney disease and reflect on a worse prognosis in cases of drug-induced nephrotoxicity. In view of the high worldwide incidence of hypovitaminosis D, the aim of this study was to investigate whether vitamin D deficiency may induce AmB/LE-related nephrotoxicity. Wistar rats were divided into four groups: control, received a standard diet for 34 days; AmB/LE, received a standard diet for 34 days and AmB/LE (5 mg/kg/day) intraperitoneally in the last 4 days; VDD, received a vitamin D-free diet for 34 days; and VDD+AmB/LE, received a vitamin D-free diet for 34 days and AmB/LE as described. At the end of the protocol, animals were euthanized and blood, urine and renal tissue samples were collected in order to evaluate AmB/LE effects on renal function and morphology. Association of AmB/LE and vitamin D deficiency led to diminished glomerular filtration rate and increased tubular injury, evidenced by reduced renal protein expression of NaPi-IIa and TRPM6 leading to hyperphosphaturia / hypermagnesuria. VDD+AmB/LE rats also presented alterations in the PTH-Klotho-FGF-23 signaling axis, urinary concentrating defect and hypertension, probably due to an inappropriate activation of the renin-angiotensin-aldosterone system. Hence, it is important to monitor vitamin D levels in AmB/LE treated patients, since vitamin D deficiency induces AmB/LE nephrotoxicity.

## Author summary

Amphotericin B (AmB) is the treatment of choice for systemic fungal infections. Despite its efficacy, clinical use of AmB has been associated with renal toxicity. In an attempt to improve the therapeutic effect and to reduce adverse reactions, lipid formulations of AmB were developed. Among these formulations, an in-house lipid emulsion preparation of AmB (AmB/LE) is a lower cost alternative with similar benefits. Furthermore, vitamin D is an essential nutrient for the regulation of several physiological activities. Hence, vitamin D deficiency or insufficiency can contribute to the progression of diseases and increase the risk of chronic illnesses as well. Nowadays, VDD is a health problem worldwide and its prevalence in general population is high, including the sunny and industrialized countries, where vitamin D supplementation has been successfully implemented. Thus, it is essential to monitor vitamin D levels in both patients treated with conventional or lipid formulations of AmB in order to ensure a better prognosis in the development of renal diseases.

## Introduction

Amphotericin B (AmB) is a macrolide polyene antibiotic frequently used for the treatment of invasive fungal infections (IFI) based on its broad-spectrum antifungal activity [1,2]. Despite its effectiveness in clinical trials, conventional AmB use is hampered by several adverse reactions and significant incidence of nephrotoxicity. Typically, AMB administration results in reduction of the glomerular filtration rate, due to renal vasoconstriction, and tubular dysfunction caused by direct interaction of the AmB with tubular cell membranes, leading to defective proximal and distal electrolyte reabsorption [3–5]. In order to reduce renal toxicity and improve both tolerability and efficacy, conventional AmB has been incorporated into phospholipid vesicles resulting in high-cost agents to resource-limited health centers [5,6]. Alternatively, studies have reported a lower cost in-house preparation of AmB as an extemporaneous lipid emulsion (AmB/LE), reducing side effects and preserving therapeutic properties [4,6].

It is well known that vitamin D deficiency (VDD) is a public health problem, affecting different regions of the world including sunny countries [7,8]. Epidemiological data indicate that low 25(OH)D levels may be partly responsible for several pathological processes, such as renin-angiotensin system activation and pro-inflammatory effects. In addition, hypovitaminosis D has been associated with the arising of hypertension, development of cardiovascular disease, diabetes mellitus and the aggravation of chronic kidney disease (CKD) [9–11]. These effects could also reflect on a worse prognosis in cases of acute kidney injury (AKI) [12,13] and drug-induced nephrotoxicity [14].

In view of the high worldwide incidence of hypovitaminosis D, the aim of this study was to investigate whether vitamin D deficiency may induce AmB/LE-related nephrotoxicity.

## Methods

### Ethics statement

The experimental procedures were developed in strict conformity with local institutional guidelines and with well-established international standards for manipulation and care of laboratory animals (Guide for the Care and Use of Laboratory Animals–NCBI–NIH), approved by the local research ethics committee (CEUA-HCFMUSP, process no. 134/15). All surgeries were performed under appropriate anesthesia, and all efforts were made to minimize suffering. The animals were anesthetized with sodium thiopental (50 mg/kg BW).

### Animals and experimental protocol

Forty-eight male Wistar rats (*Rattus novergicus*) weighing 200–250 g, obtained from the animal facilities of the University of Sao Paulo School of Medicine, were housed in standard cages and given *ad libitum* access to tap water and commercial rodent chow. Rats were randomly divided into four groups: Control (n = 12), received a standard diet for 34 days; AmB/LE (n = 12), received a standard diet for 34 days and AmB/LE (5mg/kg/day) intraperitoneally in the last 4 days; VDD (n = 12), received a vitamin D-free diet for 34 days; and VDD+AmB/LE (n = 12), received a vitamin D-free diet for 34 days and AmB/LE (5mg/kg/day) intraperitoneally in the last 4 days. The dose of AmB/LE was based on a previous study from our laboratory [4]. AmB and lipid emulsion (soy oil 200mg/mL, glycerol 25 mg/mL, egg lecithin 12 mg/mL—Lipovenos, Fresenius, Graz, Austria) were kindly provided by Hospital das Clinicas da Universidade de Sao Paulo and Fresenius, respectively.

#### Diets

Rats received a standard (Cat#960226) or vitamin D-free (Cat# 960074) diets, both obtained from *MP Biomedicals* (Irvine, CA, USA). Standard diet composition is as follows: 13.0% vitamin D-free casein, 75.0% whole wheat flour, 3.0% corn oil, 5% alphacel / nonnutritive bulk, 0.8% calcium carbonate, 5.0 IU/g diet vitamin D and 2.0% calcium phosphorus-free salt mixture. The composition of Vitamin D-free diet is similar to the standard diet, except that it does not contain vitamin D and has 0.4% Ca / P.

### Metabolic cage studies and analysis of urine samples

At the end of the protocol, rats were allocated in metabolic cages (one rat per cage), maintained on a 12-h light/dark cycle and given free access to tap water. The animals were acclimated to the housing conditions for 1 day before the experimental procedures. Twenty-four hour urine samples were collected. Urine concentrations of phosphorus, magnesium and protein were measured by a colorimetric system using commercial kits (Labtest Diagnostica, Lagoa Santa/MG, Brazil). Urinary excretions of phosphorus (U_P_V), magnesium (U_Mg_V) and protein (U_Prot_V) were determined. We determined urine osmolality (U_Osm_) with a freezing‐point osmometer (model 3D3; Advanced Instruments, Norwood, MA).

### Glomerular filtration rate

To determine glomerular filtration rate (GFR), inulin clearance studies were conducted at the end of the protocol. On day 35, the animals were anesthetized with sodium thiopental (50 mg/Kg BW) and placed on a temperature-regulated surgical table. The trachea was cannulated (PE-240 catheter) and spontaneous breathing was maintained. The jugular vein was cannulated (PE-60 catheter) for infusion of inulin and fluids. To monitor mean arterial pressure (MAP) and collect blood samples, the right carotid artery was catheterized with a PE-50 catheter. We assessed MAP with a data acquisition system (MP100; Biopac Systems, Santa Barbara, CA). To collect urine samples, the urinary bladder was cannulated (PE-240 catheter) by suprapubic incision. After completion of the cannulation surgical procedure, a loading dose of inulin (100 mg/Kg BW diluted in 1 mL of 0.9% saline) was administered through the jugular vein. Subsequently, a constant infusion of inulin (10 mg/kg BW in 0.9% saline) was started and continued at 0.04 mL/min throughout the whole experiment. Three urine samples were collected at 30-min intervals. Blood samples were obtained at the beginning and at the end of the experiment. Inulin clearance values represent the mean of three periods. Blood and urine inulin were determined by the anthrone method, and the GFR data is expressed as ml/min/100g BW [12].

### Biochemical parameters

To assess plasma levels of 25-hydroxyvitamin D [25(OH)D], parathormone (PTH), fibroblast growth factor 23 (FGF-23), aldosterone, urea, sodium (P_Na_), potassium (P_K_), phosphate (P_P_), calcium (P_Ca_) and magnesium (P_Mg_), we collected blood samples after the clearance studies. We assessed 25(OH)D, PTH, FGF-23 and aldosterone by enzyme-linked immunosorbent (ELISA) using commercial kits: 25-Hydroxyvitamin D (ALPCO, Salem, NH); Rat Intact PTH and Mouse/Rat Intact FGF-23 (Immutopics, Inc., San Clemente, CA); and Aldosterone (Enzo Life Sciences, Farmingdale, NY). P_Na_ and P_K_ were determined with specific electrodes (AVL9140 Electrolyte Analyzer, Roche Diagnostics, Risch-Rotkreuz, Switzerland). P_P_, P_Ca_ and P_Mg_ were evaluated by colorimetric assay (Labtest Diagnostica, Lagoa Santa/MG, Brazil).

### Tissue sample preparation

After the clearance experiment, we perfused kidneys with phosphate-buffered solution (PBS, pH 7.4). Fragments of right kidneys were frozen in liquid nitrogen and stored at −80°C for western blotting experiments. Fragments of left kidneys were fixed in methacarn solution (60% methanol, 30% chloroform, 10% glacial acetic acid) for 24 h and in 70% alcohol thereafter. Kidney blocks were embedded in paraffin and cut into 4-μm sections for histology.

### Total protein isolation

Kidney samples were homogenized in ice-cold isolation solution (200 mM mannitol, 80 mM HEPES and 41 mM KOH, pH 7.5) containing a protease inhibitor cocktail (Sigma Chemical Company, St. Louis, MO) in a homogenizer (PT 10/35; Brinkmann Instruments, Westbury, NY). Homogenates were centrifuged at 4000 x rpm for 30 min at 4°C to remove nuclei and cell debris. Supernatants were isolated, and protein was quantified by Bradford assay (Bio-Rad Laboratories, Hercules, CA).

### Western blot assays

For western blot analysis, 25 μg or 100 μg of total kidney protein were separated on 8%, 10% or 12% SDS-polyacrylamide minigels by electrophoresis [15]. After transfer by electroelution to PVDF membranes (GE Healthcare Limited, Little Chalfont, UK), blots were blocked for 1 h with 5% nonfat dry milk in Tris-buffered saline solution. Blots were then incubated overnight with primary antibodies for: Aquaporin 2 (AQP2, 1/1000), Sodium/phosphate cotransport type IIa (NaPi-IIa, 1/200), magnesium channel (TRPM6, 1/200), angiotensinogen (AGT, 1/100), angiotensin converting enzyme (ACE, 1/100) and α-Klotho (1/500). Primary antibodies were obtained from Santa Cruz (Santa Cruz Biotechnology, Santa Cruz, CA). The labeling was visualized with horseradish peroxidase-conjugated secondary antibody (anti-rabbit, 1:2000, or anti-goat, 1:10000; Sigma Chemical, St. Louis, MO) and enhanced chemiluminescence (ECL) detection (GE Healthcare Limited, Little Chalfont, UK). Kidney protein levels were further analyzed with a gel documentation system (Alliance 4.2; Uvitec, Cambridge, UK) and the software Image J for Windows (Image J–NIH Image). We used densitometry to quantitatively analyze the protein levels, normalizing the bands to actin expression (anti β-actin, Sigma Chemical, St. Louis, MO).

### Light microscopy

Four-mm histological sections of renal tissue were stained with hematoxylin-eosin and examined under light microscope. For the evaluation of renal damage, 40–60 grid fields (x400 magnification) measuring 0.245 mm^2^ were evaluated by graded scores according to the following criteria: (0), less than 5% of the field showing tubular epithelial swelling, vacuolar degeneration, necrosis, and desquamation; (I), 5–25% of the field presenting renal lesions; (II), involvement of 25–50% with renal damage; (III), 50–75% of damaged area; and (IV), more that 75% of the grid field presenting renal lesions. The morphometric examination was blinded to minimize observer bias, i.e. the observer was unaware of the treatment group from which the tissue originated. The mean score for each rat and the mean score for each group were calculated [13,16].

### Statistical analysis

All quantitative data were expressed as mean ± SEM (standard error of the mean). Differences among groups were analyzed with GraphPad Prism 5.0 software (GraphPad Software, La Jolla, CA) by one-way analysis of variance followed by the Student-Newman-Keuls test. Values of p < 0.05 were considered statistically significant.

## Results

As aforementioned, rats were maintained on a standard or a free-vitamin D diet for 34 days. At the end of the experimental period, VDD animals had lower levels of 25(OH)D compared to Control, demonstrating that vitamin D deficiency model was successfully achieved. Furthermore, it is important to highlight that AmB/LE treatment itself did not modify vitamin D levels (Table 1).

**Table 1:** Plasma levels of vitamin D, aldosterone, PTH and FGF-23 at the end of the experimental protocol determined in Control rats, vitamin D deficient rats (VDD), rats treated with AmB/LE 5 mg/kg/day (AmB/LE), and vitamin D deficient rats treated with AmB/LE (5 mg/kg/day).

	Control	VDD	AmB/LE	VDD+AmB/LE
25(OH)D (ng/mL)	44.3±2.4	4.3±0.4^a^	45.4±5.2^d^	4.4±0.7^a^^g^
Aldosterone (pg/mL)	1465±125	1656±72	3572±304^a^^d^	4274±345^a^^d^^i^
PTH (pg/mL)	86.5±11.2	465.8±87.1^b^	400.9±58.4^b^	406.5±112.5^c^
FGF-23 (pg/mL)	139.4±10.6	80.2±7.2^a^	115.0±12.7^f^	70.8±5.0^a^^i^

25(OH)D, 25-hydroxyvitamin D; Aldosterone, plasma aldosterone; PTH, plasma intact; FGF-23. Values are mean ± SEM.

^a^p<0.001

^b^p<0.01 and

^c^p<0.05 vs. Control

^d^p<0.001 and

^f^p<0.05 vs. VDD

^g^p<0.001 and

^i^p<0.05 vs. AmB/LE.

We did not observe any difference in body weight (BW) among the groups (Table 2), since all rats exhibited similar food ingestion, approximately 25 g/day. VDD rats exhibited a slight decrease in GFR, evidenced by diminished inulin clearance compared to Control. Animals treated with AmB/LE did not show significant changes in GFR compared to Control. However, VDD+AmB/LE group presented significantly impaired renal function compared to all experimental groups, suggesting that vitamin D deficiency may be crucial for the development of AmB-induced renal injury (Table 2). Corroborating data previously described, plasma urea concentration was higher in VDD+ AmB/LE animals compared to Control, VDD and AmB (Table 3). Another marker of renal impairment is proteinuria. VDD and VDD+AmB/LE groups presented increased urinary protein excretion compared to Control and AmB/LE (Table 3).

**Table 2:** Body weight, renal function and hemodynamic parameters at the end of the experimental protocol determined in Control rats, vitamin D deficient rats (VDD), rats treated with AmB/LE 5 mg/kg/day (AmB/LE), and vitamin D deficient rats treated with AmB/LE (5 mg/kg/day).

	Control	VDD	AmB/LE	VDD+AmB/LE
BW (g)	340±7	356±9	332±5	340±5
GFR (mL/min/100g)	0.97±0.04	0.79±0.02^c^	0.99±0.06^f^	0.55±0.04^a^^e^^g^
MAP (mmHg)	123±3	130±4	131±5	148±5^b^^f^^i^

BW, body weight, GFR, inulin clearance; MAP, mean arterial pressure. Values are mean ± SEM.

^a^p<0.001

^b^p<0.01 and

^c^p<0.05 vs. Control

^e^p<0.01 and

^f^p<0.05 vs. VDD

^g^p<0.001 and

^i^p<0.05 vs. AmB/LE.

**Table 3:** Biochemical parameters at the end of the experimental protocol determined in Control rats, vitamin D deficient rats (VDD), rats treated with AmB/LE 5 mg/kg/day (AmB/LE), and vitamin D deficient rats treated with AmB/LE (5 mg/kg/day).

	Control	VDD	AmB/LE	VDD+AmB/LE
P_Na_ (mmol/L)	138±1	139±1	141±1	140±1
P_K_ (mmol/L)	4.5±0.1	4.7±0.1	4.1±0.3	4.4±0.1
P_Ca_ (mmol/L)	1.20±0.02	0.94±0.03^a^	1.17±0.02^d^	0.96±0.04^a^^g^
P_P_ (mg/dL)	8.3±0.3	6.4±0.3^a^	5.5±0.4^a^	4.6±0.3^a^^e^^i^
P_Mg_ (mg/dL)	2.01±0.03	1.98±0.06	1.85±0.04	1.99±0.03
P_Ur_ (mg/dL)	49±1	51±4	77±9	152±28^a^^d^^h^
U_P_V (mg/day)	16.8±1.4	23.9±1.5^b^	21.7±1.0^c^	20.9±1.3^c^
U_Mg_V (mg/day)	1.9±0.2	1.8±0.1	2.0±0.1	3.2±0.3^a^^d^^g^
UV (mL)	17±1	26±1^b^	24±1^b^	32±2^a^^f^^h^
U_Osm_ (mOsm/kg/H_2_O)	1028±89	656±64^a^	635±32^a^	367±21^a^^d^^g^
U_Prot_V (mg/day)	7.5±0.5	13.1±0.5^a^	9.6±0.7^e^	12.7±1.0^a^^h^

P_Na_, plasma sodium concentration; P_K_, Plasma potassium concentration; P_Ca_, plasma calcium concentration; P_P_, plasma phosphate concentration, P_Mg_, plasma magnesium concentration, P_Ur_, plasma urea concentration, U_P_V; urine excretion of phosphorus; U_Mg_V, urine excretion of magnesium; UV, urinary volume; UOsm, urinary osmolality; U_Prot_V, proteinuria (mg/dia). Values are mean ± SEM.

^a^p<0.001

^b^p<0.01 and

^c^p<0.05 vs. Control

^d^p<0.001

^e^p<0.01 and

^f^p<0.05 vs. VDD

^g^p<0.001

^h^p<0.01 and

^i^p<0.05 vs. AmB/LE.

**Fig 1:**
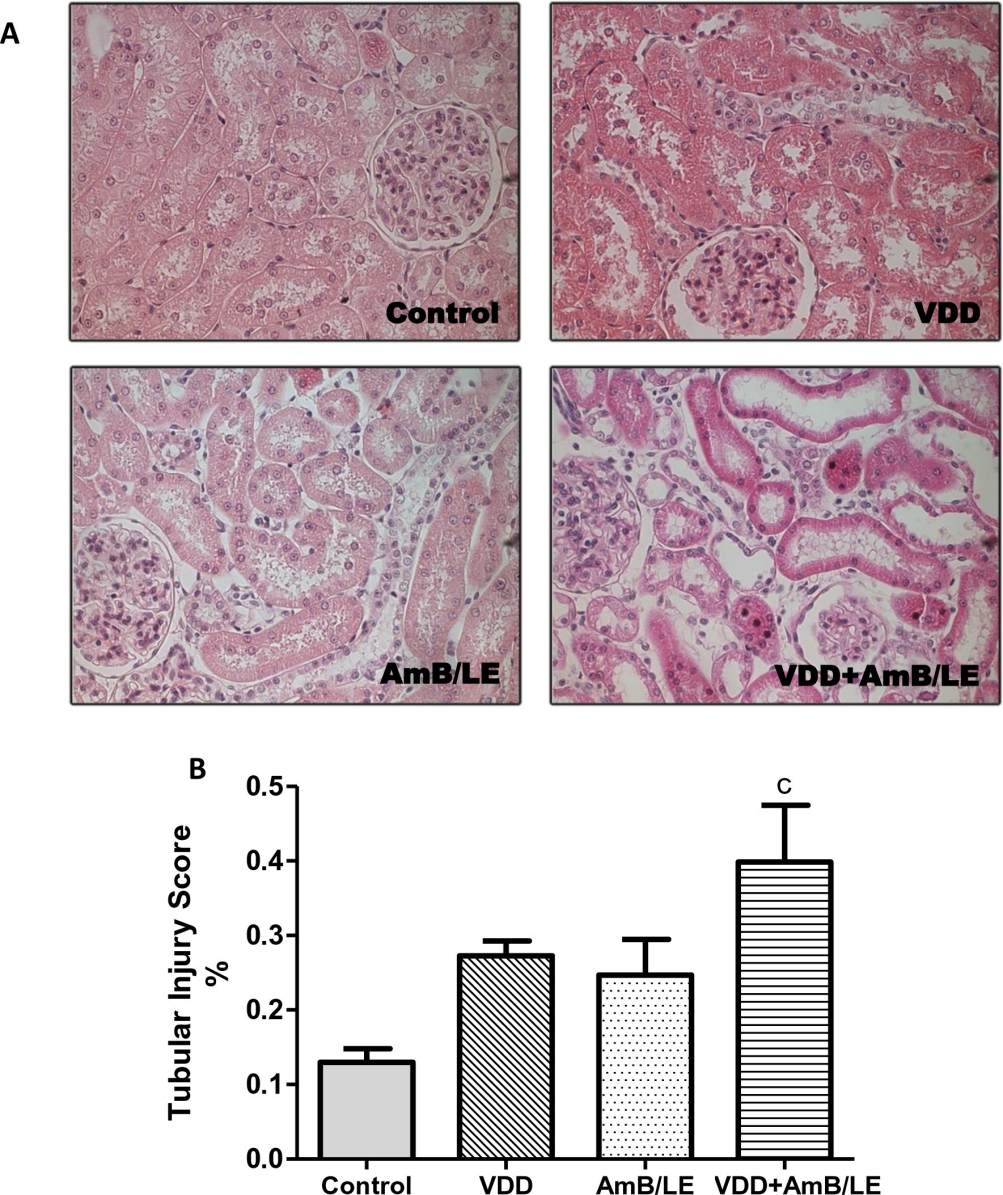
Tubular injury score. (A) Representative photomicrographs of tubular injury in Control, VDD, AmB/LE and VDD+AmB/LE rats. Magnification, 400x. (B) Bar graph of tubular injury score. Data are mean ± SEM. ^c^p<0.05 vs. Control. (VDD, vitamin D deficiency; AmB/LE, Amphotericin B lipid emulsion; VDD+AmB/LE, vitamin D deficiency + Amphotericin B lipid emulsion).

Our histological study revealed that both VDD (0.31±0.04) and AmB/LE (0.25±0.05) groups did not show significant renal tubular injury compared to Control (0.13±0.02). Vitamin D deficient rats treated with AmB/LE (0.40±0.08) exhibited higher tubular injury score compared to all experimental groups. VDD+AmB/LE histological alterations included areas of denuded basement membrane, tubular cell necrosis, flattening of proximal tubular cells with brush border loss and tubular atrophy or dilatation (Fig 1A and 1B). It is important to point out that these alterations were focal and slight. However, the development of tubular injury may indicate that vitamin D deficiency could be responsible for the nephrotoxic effects of AmB/LE.

**Fig 2:**
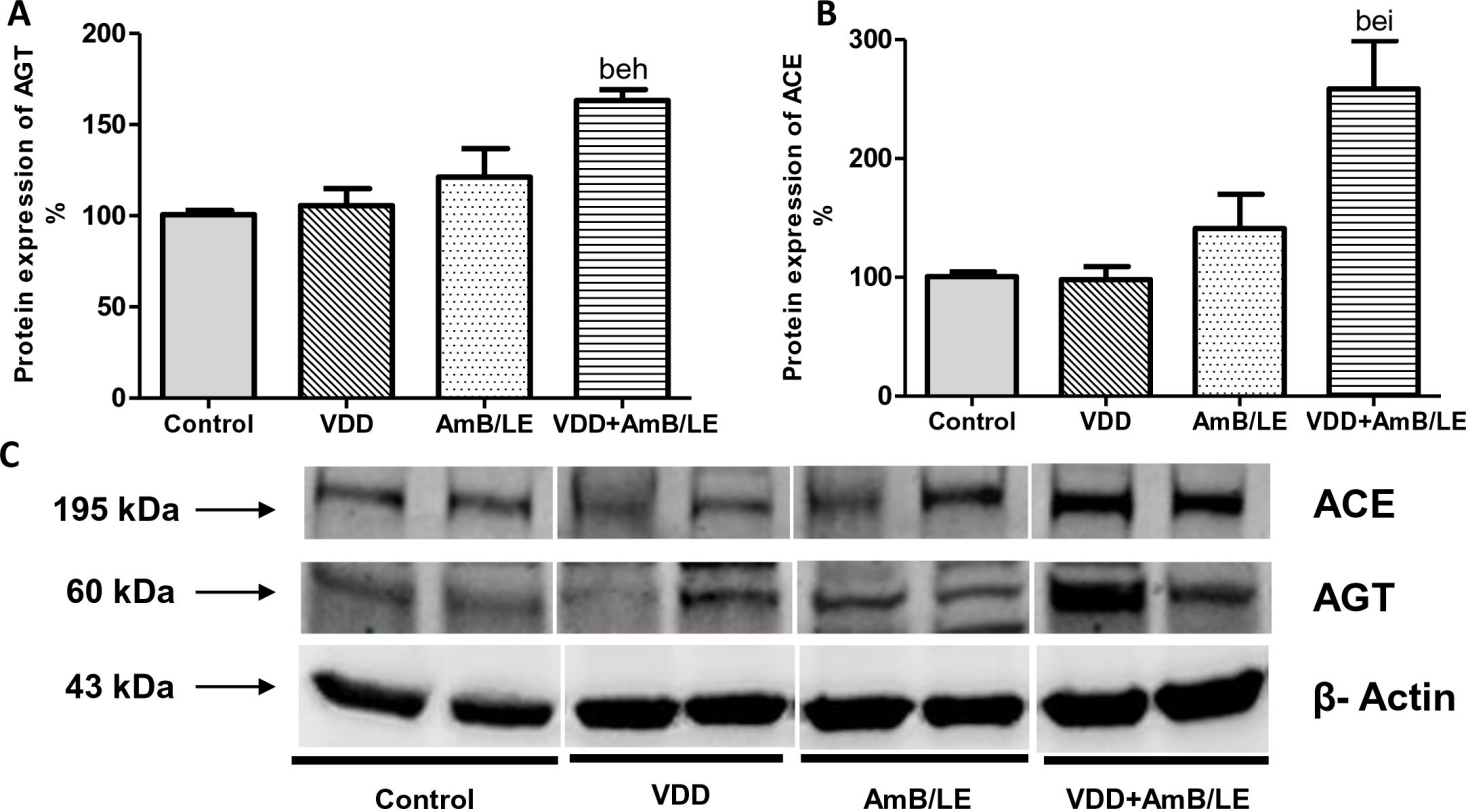
Semiquantitative immunoblotting of kidney fractions for angiotensinogen (AGT) and angiotensin converting enzyme (ACE). (A) Densitometric analysis of AGT protein expression from Control, VDD, AmB/LE and VDD+AmB/LE rats. (B) Densitometric analysis of ACE protein expression from Control, VDD, AmB/LE and VDD+AmB/LE rats. (C) Immunoblots reacted with anti-AGT and anti-ACE revealing a 60 kDa and 195 kDa bands, respectively. Data are mean ± SEM. ^b^p<0.01 vs. Control; ^e^p<0.01 vs. VDD; ^h^p<0.01 and ^i^p<0.05 vs. AmB/LE. (VDD, vitamin D deficiency; AmB/LE, Amphotericin B lipid emulsion; VDD+AmB/LE, vitamin D deficiency + Amphotericin B lipid emulsion).

In addition to impaired renal function, VDD+AmB/LE animals also presented higher MAP compared to groups C, VDD and AmB/LE (Table 2). Supporting data above-mentioned, renal protein expression of AGT (163±6%) and ACE (258±40%) were higher in VDD+AmB/LE animals compared to Control (100±2% for AGT and 100±4% for ACE), VDD (105±9% for AGT and 98±11% for ACE) and AmB/LE (121±15% for AGT and 141±28% for ACE) groups (Fig 2A–2C). Furthermore, both groups treated with AmB/LE showed higher plasma aldosterone concentration. However, VDD+AmB/LE animals exhibited an even greater increase in this parameter compared to Control, VDD and AmB/LE (Table 1). Altogether, these data suggest a possible involvement of the Renin-Angiotensin-Aldosterone System (RAAS) in the arising of hypertension in VDD+AmB/LE rats.

Plasma levels of sodium and potassium did not change among the experimental groups (Table 3). As expected, plasma phosphate and calcium concentrations were lower in VDD groups, since diet composition has lower concentrations of calcium and phosphorus (0.4% Ca and 0.4% P) compared to standard diet. Treatment with AmB/LE alone also led to decreased plasma phosphate levels. Combination of VDD and AmB/LE aggravated hypophosphatemia (Table 3).

**Fig 3:**
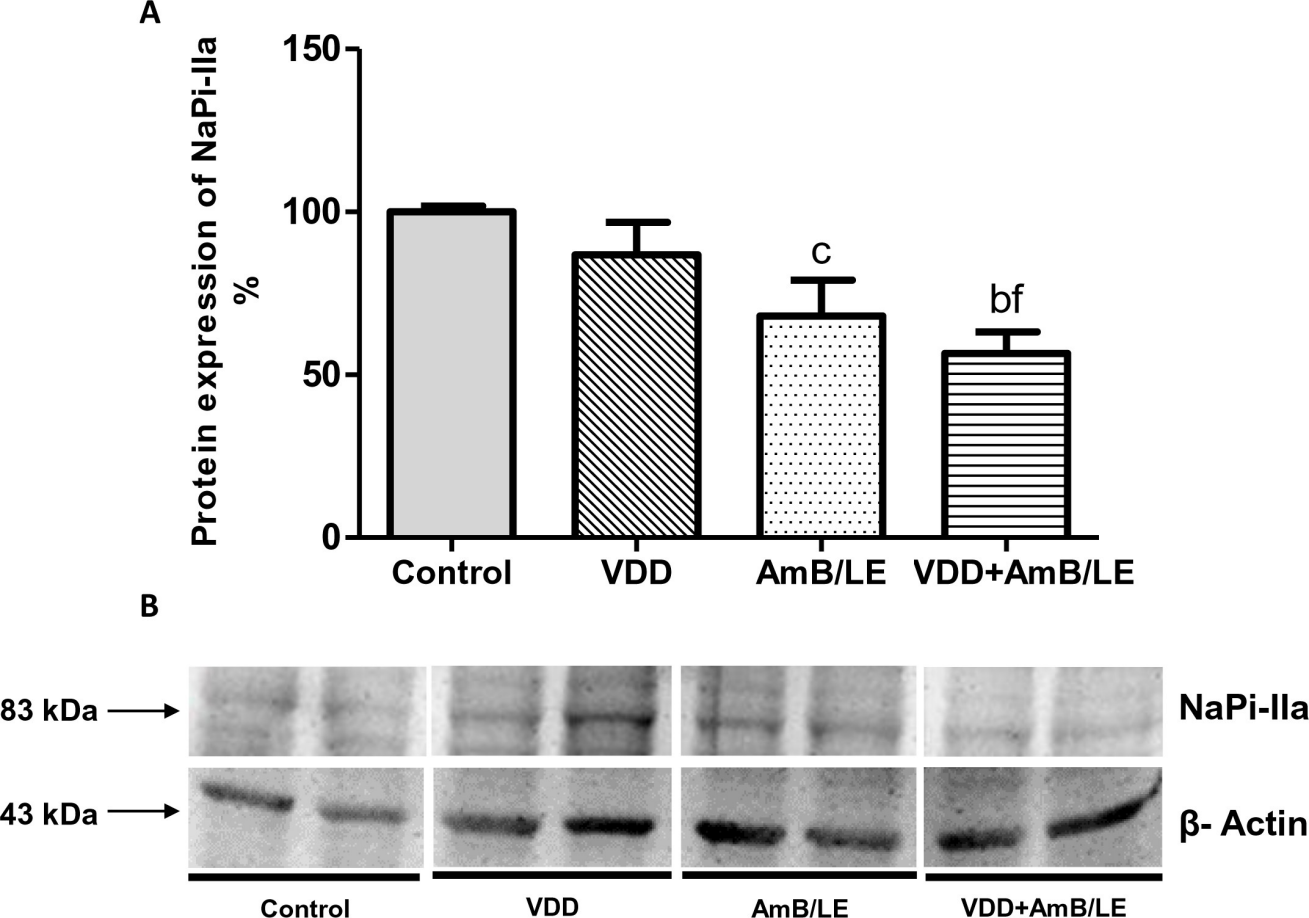
Semiquantitative immunoblotting of kidney fractions for NaPi-IIa transporter. (A) Densitometric analysis of samples from Control, VDD, AmB/LE and VDD+AmB/LE rats. (B) Immunoblots reacted with anti-NaPi-IIa revealing a 83 kDa band. Data are mean ± SEM. ^b^p<0.01 and ^c^p<0.05 vs. Control; ^f^p<0.05 vs. VDD (VDD, vitamin D deficiency; AmB/LE, Amphotericin B lipid emulsion; VDD+AmB/LE, vitamin D deficiency + Amphotericin B lipid emulsion).

**Fig 4:**
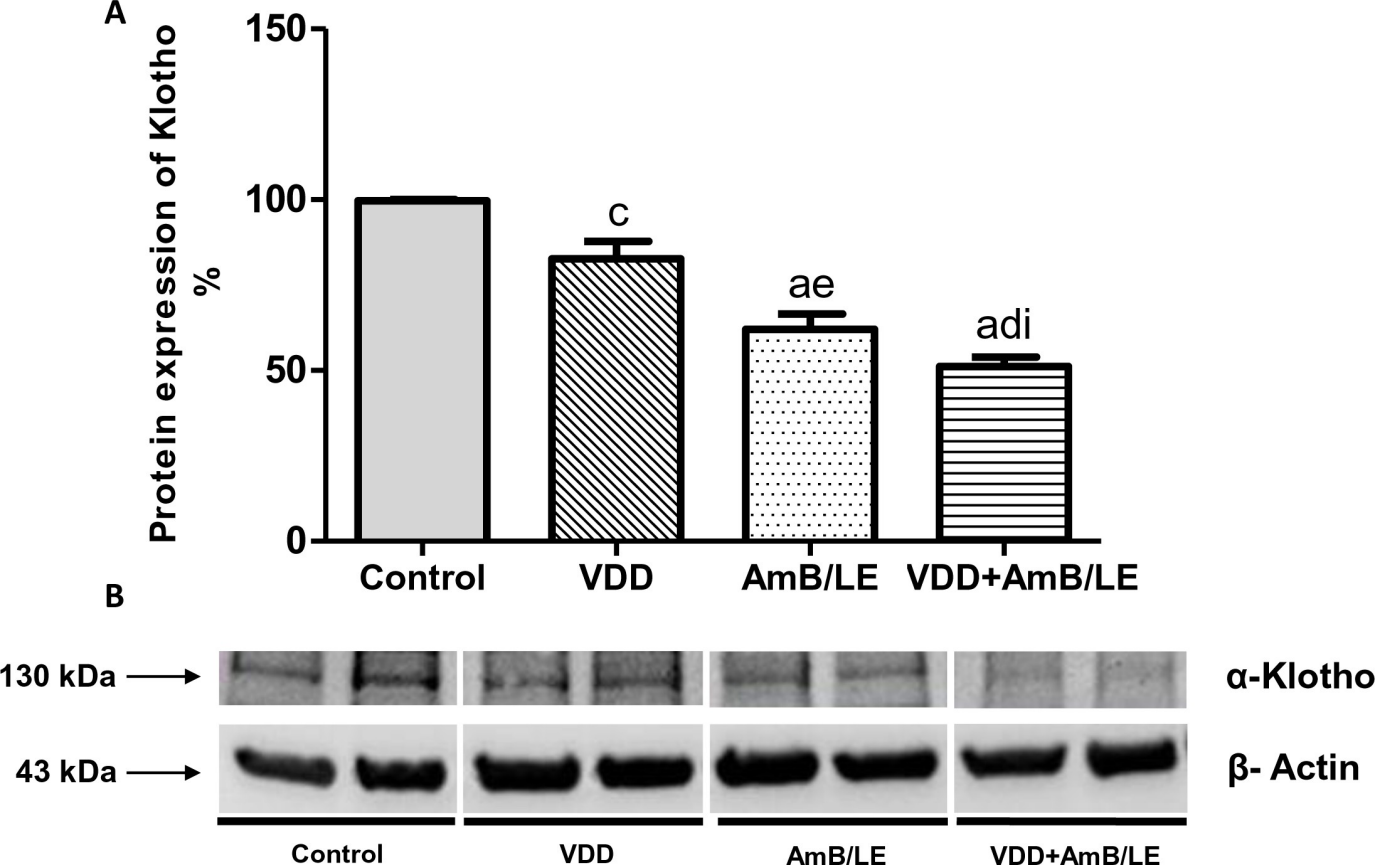
Semiquantitative immunoblotting of kidney fractions for Klotho. (A) Densitometric analysis of samples from Control, VDD, AmB/LE and VDD+AmB/LE rats. (B) Immunoblots reacted with anti-α-Klotho revealing a 130 kDa band. Data are mean ± SEM. ^a^p<0.001 and ^c^p<0.05 vs. Control; ^d^p<0.001 and ^e^p<0.01 vs. VDD; ^i^p<0.05 vs. AmB/LE (VDD, vitamin D deficiency; AmB/LE, Amphotericin B lipid emulsion; VDD+AmB/LE, vitamin D deficiency + Amphotericin B lipid emulsion).

Proximal tubule function was impaired in VDD, AmB/LE and AmB/LE groups compared to Control, evidenced by higher urinary excretion of phosphorus (Table 3). In addition, renal protein expression of NaPi-IIa was diminished by approximately 30% in AmB/LE animals and 40% in VDD+AmB/LE rats compared to Control (Fig 3A and 3B), indicating that hyperphosphaturia is possibly related to lower sodium/phosphate cotransporter expression. Moreover, VDD, AmB/LE and VDD+AmB/LE groups presented an increased plasma PTH concentration compared to Control (Table 1). The evaluation of plasma concentration of FGF-23 revealed lower levels of this hormone in vitamin D deficient rats compared to Control and AmB/LE groups (Table 1). In order to further investigate PTH-Klotho-FGF-23 axis, we determined renal protein expression of α-Klotho. α-Klotho protein expression was significantly reduced in VDD (83±5%), AmB/LE (62±4%) and VDD+AmB/LE (51±2%) groups compared to Control (100±1%) (Fig 4A and 4B). Our results showed that not only VDD but also AMB/LE led to substantial changes in PTH-Klotho-FGF-23 axis, suggesting that such imbalance may contribute to the progression of AmB/LE-induced nephrotoxicity.

**Fig 5:**
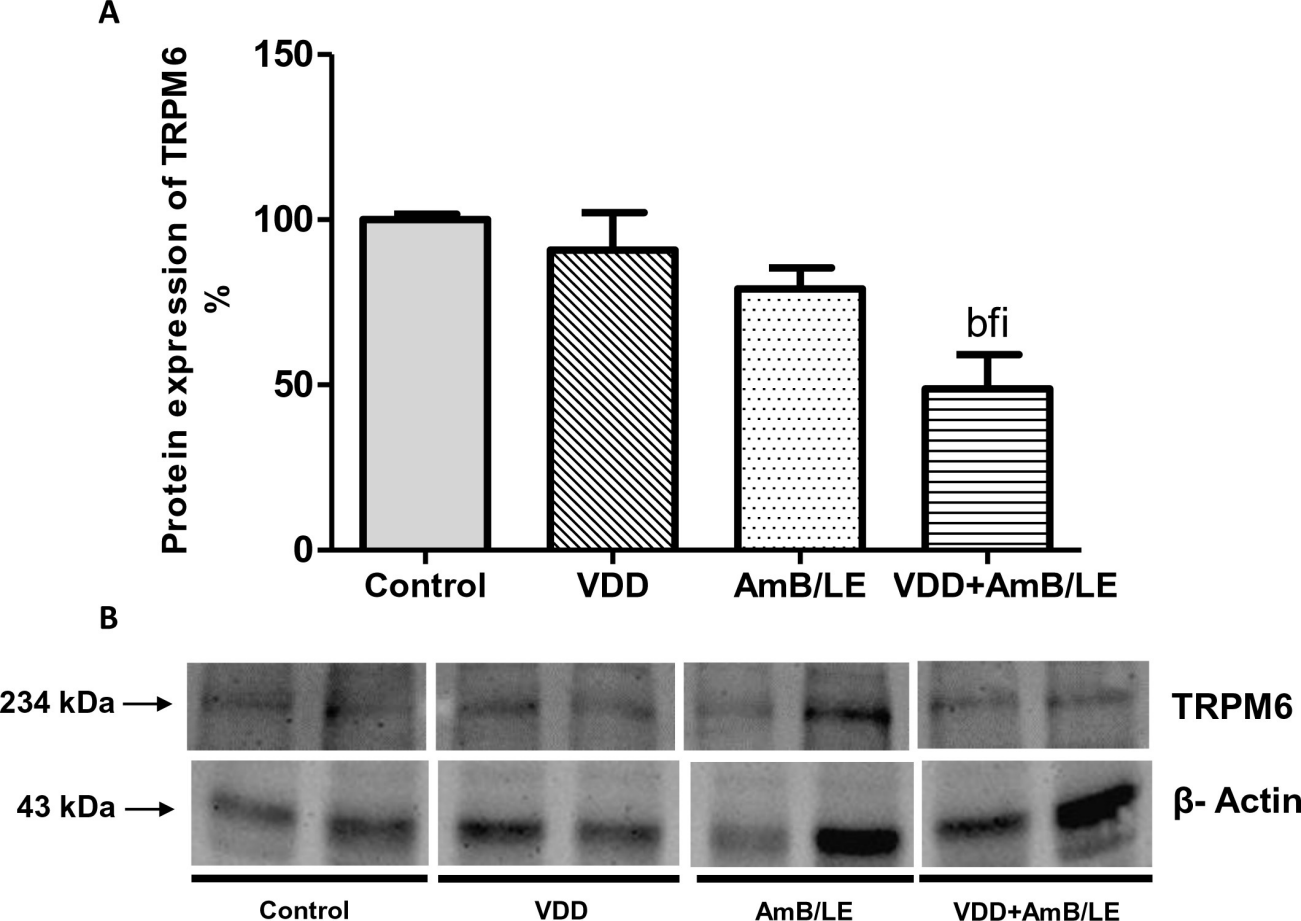
Semiquantitative immunoblotting of kidney fractions for TRPM6 channel. (A) Densitometric analysis of samples from Control, VDD, AmB/LE and VDD+AmB/LE rats. (B) Immunoblots reacted with anti-TRPM6 revealing a 234 kDa band. Data are mean ± SEM. ^b^p<0.01 vs. Control; ^f^p<0.05 vs. VDD; ^i^p<0.05 vs. AmB/LE (VDD, vitamin D deficiency; AmB/LE, Amphotericin B lipid emulsion; VDD+AmB/LE, vitamin D deficiency + Amphotericin B lipid emulsion).

Plasma magnesium concentration did not change among the experimental groups (Table 3). Nevertheless, vitamin D deficient animals treated with AmB/LE exhibited higher urine excretion of magnesium compared to Control, VDD and AmB/LE (Table 3). Likewise, only VDD+AmB/LE rats (49±11%) showed significant lower renal protein expression of TRPM6 compared to Control (100±2%), VDD (91±12%) and AmB/LE (79±6%) (Fig 5A and 5B), indicating a possible involvement of this channel in the development of hypermagnesuria in vitamin D deficient animals treated with AmB/LE.

**Fig 6:**
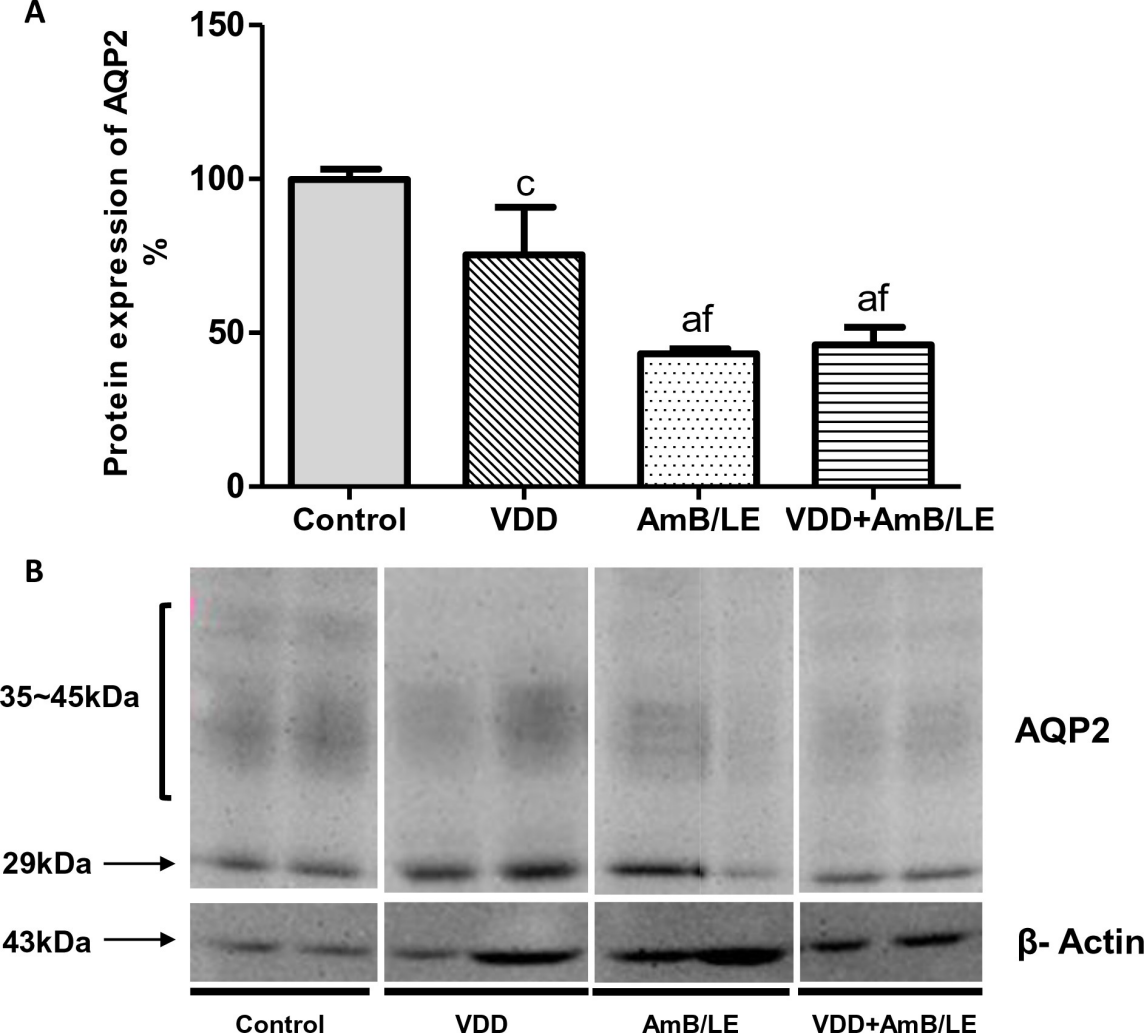
Semiquantitative immunoblotting of kidney fractions for Aquaporin 2 channel. (A) Densitometric analysis of samples from Control, VDD, AmB/LE and VDD+AmB/LE rats. (B) Immunoblots reacted with anti-AQP2 revealing both 29 and 34~45 kDa bands. Data are mean ± SEM. ^a^p<0.001 and ^c^p<0.05 vs. Control; ^f^p<0.05 vs. VDD (VDD, vitamin D deficiency; AmB/LE, Amphotericin B lipid emulsion; VDD+AmB/LE, vitamin D deficiency + Amphotericin B lipid emulsion).

Association of vitamin D deficiency and treatment with AmB/LE resulted in a significant increase in urine volume and a subsequent decrease in urine osmolality compared to Control, VDD and AmB/LE. VDD and AmB/LE groups also presented a slight increase in urinary volume and a lower urine osmolality compared to Control (Table 3). These alterations were accompanied by a diminished renal expression of AQP2 in groups AmB/LE (43±2%) and VDD+AmB/LE (46±6%) compared to Control (100±3%) and VDD (75±16%) (Fig 6A and 6B).

## Discussion

AmB is the drug of choice for the treatment of IFI, however clinical use of AmB has been associated with renal toxicity. The high rate of nephrotoxicity has enabled the development of modified AmBs [4,6]. Among these formulations, an extemporaneous lipid emulsion preparation of AmB is a lower cost alternative with similar benefits [4,6]. Moreover, studies have been showing a high prevalence of VDD in general population, reflecting on a worse prognosis in cases of acute kidney injury and drug-induced nephrotoxicity [7,10,14]. In this study, we showed that association of VDD and AmB/LE led to impaired renal function, hypertension and urinary concentrating defect.

Our data demonstrated that vitamin D deficient animals treated with AmB/LE presented impaired renal function, evidenced by lower GFR, higher plasma urea concentration, hyperphosphaturia, hypermagnesuria, proteinuria and mild tubular injury. It is well known that AmB-induced nephrotoxicity is due to the interaction of AmB with sterols from cell membranes, resulting in pore formation, defected electrolyte flux and loss of cell viability [3,17,18]. Furthermore, experimental and clinical trials have reported that treatment with AmB leads to denuded basement membrane, intratubular casts and tubular cell necrosis. These studies also described no severe morphological glomerular damage [19,20]. However, a previous study from our laboratory reported that the association of AmB and lipid emulsion efficiently reduced renal toxicity [4]. On this hand, our results also showed that AmB/LE did not change either GFR or renal morphology, suggesting that vitamin D deficiency may play an important role in the development of renal injury in VDD+AmB/LE rats.

In this study, VDD+AmB/LE rats developed hypertension, accompanied by a notable increase in renal protein expression of AGT and ACE, and higher levels of plasma aldosterone. According to Meaudre *et al*., AmB acts directly on vascular smooth muscle cells resulting in local vasoconstriction and subsequent systemic higher blood pressure [21]. However, the mechanisms involved in the arising of hypertension in AmB-treated patients are still unclear [21]. On the other hand, it is well known that vitamin D is a negative endocrine regulator of the RAAS and the unappropriated activation of this system has been related to hypertension [22,23]. Furthermore, previous studies have demonstrated that VDD regulates blood pressure through direct effects on the vascular endothelium, promoting hypertension [10,12]. Thus, it is possible to speculate that VDD associated with AmB/LE treatment may have exacerbated arterial hypertension observed in this group.

As expected, VDD animals exhibited hypophosphatemia, hypocalcemia and increased plasma PTH concentration, since vitamin D deficiency diminishes calcium intestinal absorption, resulting in decreased calcium concentration and increased production of PTH [14]. Interestingly, higher PTH has been associated with the development of hypertension because of its effects on vascular smooth muscle cells, increasing vascular tone and arterial blood pressure [24,25]. In addition, AmB/LE treatment itself led to reduced levels of plasma phosphate and the association of VDD with AmB/LE significantly increased hypophosphatemia. Lower plasma phosphate concentration was accompanied by increased phosphaturia and decreased renal protein expression of NaPi-IIa cotransporter in AmB/EL and VDD+AmB/LE groups, characterizing a proximal tubular injury. It is important to point out that PTH also induces phosphaturia and treatment with calcitriol decreases urinary excretion of phosphorus in an experimental rat model, indicating that vitamin D may stimulate renal phosphate transport [26,27].

Moreover, Razzaque *et al*. reported that, in addition to PTH and calcitriol, FGF23 may directly or indirectly downregulate NaPi activity, leading to reduced reabsorption of phosphate [28]. In our study, we found diminished plasma FGF-23 concentration in VDD and VDD+AmB/LE groups. Although FGF-23 is an early biomarker in the development of chronic kidney disease (CKD), this may occur as a compensatory response, in order to restore vitamin D levels and hyperphosphaturic effect [12]. Supporting our findings, previous experimental studies showed that rats under VDD and CKD progression also presented lower levels of FGF-23 [10,12]. Recent reports have been suggesting an interesting link between phosphate and FGF-23/Klotho axis. Traditionally, Klotho is related to aging processes in mammals and its deficiency is considered the initiator of CKD-related mineral disorders [29,30]. VDD animals exhibited a slight decrease in renal protein expression of Klotho compared to Control. Surprisingly, AmB/LE treated rats also showed a lower renal protein expression of Klotho compared to Control and VDD groups. Association of VDD and AmB/LE exacerbated Klotho deficiency. Altogether, these results indicate that disturbances in PTH-Klotho-FGF-23 axis might be responsible for the aggravation of AmB/LE-induced nephrotoxicity.

Clinical trials have reported that conventional AmB is more likely to induce renal magnesium wasting and mild hypomagnesemia than lipid formulations [5,31]. Corroborating these data previously shown, our lipid formulation of AmB did not change urinary excretion of magnesium. However, the association of VDD and AmB/LE led to hypermagnesuria. Renal magnesium loss can be due to polyuria or tubular reabsorption defect [32]. Higher urinary excretion of magnesium was accompanied by reduced renal protein expression of TRPM6 in VDD+AmB/LE rats. TRPM6 is the major channel related to magnesium handling and contributes to magnesium reabsorption across distal convoluted tubule [33]. In our study, VDD possibly played an important role in the management of magnesium in the renal tubule and in the onset of hypermagnesuria in vitamin D deficient animals treated with AmB/LE.

It is well-established that both VDD and AmB/LE led to an impaired renal concentrating ability, evidenced by higher 24h-urine volume and decreased urinary osmolality [4,13]. Indeed, our results showed that VDD and AmB/LE groups exhibited increased 24h-urine volume and diminished urinary osmolality compared to Control. In addition, renal protein expression of AQP2 was decreased in VDD and AmB/LE rats compared to Control. Corroborating our data, previous experimental studies described that treatment with AmB alone inhibits the AVP/V2R signaling pathway, resulting in diminished water reabsorption via AQP2 in the collecting duct of the kidney [34,35]. Combination of VDD and AmB/LE resulted in a more severe polyuria associated with lower protein expression of AQP2, suggesting that VDD might have exacerbated renal concentrating defect in AmB/LE-induced renal toxicity.

In conclusion, our results confirm that vitamin D deficiency induces AmB/LE nephrotoxicity possibly due to impaired renal function accompanied by tubular injury, the arising of hypertension, alterations in the PTH-Klotho-FGF-23 signaling axis and water balance dysfunction. It is worth mentioning that an in-house lipid emulsion preparation of AmB preserves therapeutic properties and is not as expensive as pharmaceutical lipid formulations. Thus, it is essential to monitor vitamin D levels in both patients treated with conventional or lipid formulations of AmB, in order to ensure a better prognosis in the development of renal diseases.
